# Apoptosis Signal-Regulating Kinase 1 Is Involved in WISP-1–Promoted Cell Motility in Human Oral Squamous Cell Carcinoma Cells

**DOI:** 10.1371/journal.pone.0078022

**Published:** 2013-10-21

**Authors:** Jing-Yuan Chuang, An-Chen Chang, I-Ping Chiang, Ming-Hsui Tsai, Chih-Hsin Tang

**Affiliations:** 1 Department of Medical Laboratory Science and Biotechnology, China Medical University, Taichung, Taiwan; 2 Department of Pathology, China Medical University Hospital, Taichung, Taiwan; 3 Department of Otolaryngology, China Medical University Hospital, Taichung, Taiwan; 4 Graduate Institute of Basic Medical Science, China Medical University, Taichung, Taiwan; 5 Department of Pharmacology, School of Medicine, China Medical University, Taichung, Taiwan; 6 Department of Biotechnology, College of Health Science, Asia University, Taichung, Taiwan; UAE University, Faculty of Medicine & Health Sciences, United Arab Emirates

## Abstract

Oral squamous cell carcinoma (OSCC) has a tendency to migrate and metastasize. WNT1-inducible signaling pathway protein 1 (WISP-1) is a cysteine-rich protein that belongs to the Cyr61, CTGF, Nov (CCN) family of matrix cellular proteins. The effect of WISP-1 on human OSCC cells, however, is unknown. Here, we showed that WISP-1 increased cell migration and intercellular adhesion molecule-1 (ICAM-1) expression in OSCC cells. Pretreatment of cells with integrin αvβ3 monoclonal antibody (mAb) significantly abolished WISP-1–induced cell migration and ICAM-1 expression. On the other hand, WISP-1–mediated cell motility and ICAM-1 upregulation were attenuated by ASK1, JNK, and p38 inhibitor. Furthermore, WISP-1 also enhanced activator protein 1 (AP-1) activation, and the integrin αvβ3 mAb, and ASK1, JNK, and p38 inhibitors reduced WISP-1–mediated AP-1 activation. Moreover, WISP-1 and ICAM-1 expression correlated with the tumor stage of patients with OSCC. Our results indicate that WISP-1 enhances the migration of OSCC cells by increasing ICAM-1 expression through the αvβ3 integrin receptor and the ASK1, JNK/p38, and AP-1 signal transduction pathways.

## Introduction

Oral squamous cell carcinoma (OSCC) represents 1–2% of all human malignancies. It is the most common head and neck cancer and is characterized by poor prognosis and low survival rate. OSCC has been reported to migrate into maxillary and mandibular bones [1] and have a potent capacity to invade locally and metastasize distantly [2], [3]. Hence, decrease in its ability to invade and metastasize may facilitate the development of effective adjuvant therapy.

WNT1-inducible signaling pathway protein 1 (WISP-1) is a cysteine-rich protein that belongs to the Cyr61, CTGF, Nov (CCN) family of matricellular proteins, which have developmental functions [4], [5]. CCN family proteins are mostly secreted and are associated to the extracellular matrix (ECM) which has been demonstrated to play important roles in tumor development, including tumor survival, proliferation, migration, and invasion [6], [7]. They may connect signaling pathways and facilitate crosstalk between the epithelium and stroma [4]. It has been reported that overexpression of WISP-1 in normal rat kidney fibroblasts promotes their transformation [8]. On the other hand, WISP-1 is expressed in the developing breast tumors in transgenic mice [9]. Moreover, increasing evidence suggests that WISP-1 enhanced tumorigenesis and metastasis in many types of cancer [10], [11]. These data suggest that WISP-1 plays a critical role during cancer development and metastasis.

Tumor invasion and metastasis are the main biological characteristics of cancer cells. Metastasis is the major cause of cancer death and involves multiple processes including invading cells change the cell-cell adhesion properties, rearrange the ECM environment, suppress anoikis, and reorganize their cytoskeletons [12]. There are several cell adhesion molecules have been reported to be involved in tumor progression and metastasis such as integrin, cadherin, and immunoglobulin superfamilies [13], [14]. Intercellular adhesion molecule-1 (ICAM-1, also called CD54), a member of the immunoglobulin supergene family, is an inducible surface glycoprotein that mediates adhesion-dependent cell-to-cell interactions [15], [16]. ICAM-1 has been reported to mediate the migration of leukocytes from the capillary bed into the tissue [17]. On the other hand, ICAM-1 also promotes the movement of cells through the ECM [17]. Recently study indicated that ICAM-1 plays an important role during lung cancer invasion [18]. Pretreatment with ICAM-1 antibody or transfection with antisense ICAM-1 has been reported to reduce the migration of breast cancer cells [19]. Therefore, ICAM-1 might play a critical role in tumorigenesis, and its disruption may prevent metastasis.

Apoptosis signal-regulating kinase 1 (ASK1) is a member of the MAPK kinase kinase (MKKK) family. It activates the c-jun N-terminal kinase (JNK) and p38 signaling pathways; affects multiple cellular functions [20], including survival, differentiation, and the innate immune response [21], [22], [23], and has been reported to regulate vascular smooth muscle cell migration [24]. In addition, ASK1 plays a crucial role in regulating tumor metastasis [25]. However, the ASK1 activation in cell migration and ICAM-1 expression in human OSCC is largely unknown. In this study, we explored the intracellular ASK1 signaling pathway involved in WISP-1–induced ICAM-1 production and cell migration in human OSCC. The results show that WISP-1 binds αvβ3 integrin and causes the activation of the ASK1, JNK/p38, and AP-1 pathways, which upregulates ICAM-1 expression and promotes the migration of human OSCC cells. In addition, the high level of WISP-1 expression correlated strongly with ICAM-1 expression and tumor stage. Our results indicate that WISP-1 is a crucial factor during the metastasis of OSCC cells.

## Materials and Methods

### Materials

Protein A/G beads; anti-mouse and anti-rabbit IgG-conjugated horseradish peroxidase; rabbit polyclonal antibodies specific for ICAM-1, WISP-1, ASK1, p-p38, p38, p-JNK, JNK, p-c-Jun, c-Jun, and β-actin; and the small interfering RNA (siRNA) against ICAM-1, c-Jun, ASK1 short hairpin RNA (shRNA), WISP-1 shRNA, and control shRNA plasmids were purchased from Santa Cruz Biotechnology (Santa Cruz, CA, USA). Rabbit polyclonal antibody specific for ASK1 that is phosphorylated at Thr^845^ was purchased from Cell Signaling and Neuroscience (Danvers, MA, USA). The recombinant human WISP-1 was purchased from R&D Systems (Minneapolis, MN, USA). The p38 dominant negative mutant was provided by Dr. J. Han (University of Texas Southwestern Medical Center, Dallas, TX, USA). The JNK dominant negative mutant was provided by Dr. M. Karin (University of California, San Diego, CA, USA). The luciferase assay kit was purchased from Promega (Madison, WI, USA). All other chemicals were purchased from Sigma-Aldrich (St. Louis, MO, USA).

### Cell culture

The human OSCC cell lines (SCC4 and CAL-27 cells) were purchased from the American Type Culture Collection (Rockville, MD, USA). The cells were maintained in Dulbecco’s modified eagle medium (DMEM) supplemented with 20 mM HEPES and 10% heat-inactivated fetal calf serum (FCS), 2 mM glutamine, penicillin (100 U/mL), and streptomycin (100 µg/mL) at 37°C with 5% CO_2_.

The WISP-1 shRNA-expressing cells were selected with puromycin. Surviving cells were picked and expanded to prepare clonal cell populations. For monolayer growth curves, 10^4^ cells were plated in 6-well plates and grown for 1 to 6 days. Cells were trypsinized, and cell numbers were counted [26]. Cells were cultured in DMEM supplemented with 20 mM HEPES and 10% heat-inactivated FCS, 2 mM glutamine, penicillin (100 U/mL), and streptomycin (100 µg/mL) at 37°C with 5% CO_2_.

### Migration and invasion assay

The migration assay was performed using Transwell inserts (Costar, New York, NY, USA; pore size, 8 µm) in 24-well dishes. For invasion assays, filters were precoated with 30 µL of Matrigel basement membrane matrix (BD Biosciences, Bedford, MA, USA) for 30 min. The following procedures were the same for both migration and invasion assays. Before the migration assay was performed, cells were pretreated for 30 min with different concentrations of inhibitors, including thioredoxin, SP600125, SB203580, and vehicle control (0.1% dimethyl sulfoxide [DMSO]). Approximately 1 × 10^4^ cells in 200 µL of serum-free medium were placed in the upper chamber, and 300 μL of the serum-free medium containing WISP-1 was placed in the lower chamber. The plates were incubated for 24 h at 37°C in 5% CO_2_. Then, the cells were fixed in 3.7% formaldehyde solution for 15 min and stained with 0.05% crystal violet in phosphate-buffered saline (PBS) for 15 min. Cells on the upper side of the filters were removed with cotton-tipped swabs, and the filters were washed with PBS. Cells on the underside of the filters were examined and counted under a microscope. Each clone was plated in triplicate in each experiment, and each experiment was repeated at least 3 times. The number of migrating cells in each experiment was adjusted by the cell viability assay to correct for proliferation effects of WISP-1 treatment (corrected migrating cell number  =  counted migrating cell number/percentage of viable cells) [27].

### Quantitative real-time polymerase chain reaction (PCR)

Total RNA was extracted from OSCC cells using a TRIzol kit (Invitrogen Carlsbad, CA, USA). The reverse transcription reaction was performed using 2 µg of total RNA that was reverse transcribed into cDNA using oligo(dT) primer [28], [29]. The quantitative real-time PCR (qPCR) analysis was carried out using Taqman® one-step PCR Master Mix (Applied Biosystems, CA, USA). Two microliters of total cDNA mixtures was added per 25-µL reaction with sequence-specific primers and Taqman® probes. All target gene primers and probes were purchased commercially (β-actin was used as an internal control) (Applied Biosystems). qPCR assays were performed in triplicate (1 independent RNA sample per treatment) on a StepOnePlus sequence detection system. The cycling conditions were as follows: polymerase activation at 95°C for 10 min followed by 40 cycles at 95°C for 15 s and 60°C for 60 s. The threshold was set above the no-template control background and within the linear phase of the target gene amplification to calculate the cycle number at which the transcript was detected (denoted C_T_).

### Western blot analysis

Cellular lysates were prepared as described [30], [31]. Proteins were resolved by sodium dodecyl sulfate polyacrylamide gel electrophoresis (SDS-PAGE) and transferred to Immobilon polyvinyldifluoride (PVDF) membranes. The blots were blocked with 4% bovine serum albumin (BSA) for 1 h at room temperature and then probed with rabbit anti-human antibodies against ICAM-1, p-p38, p38, p-JNK, JNK, p-c-Jun, or c-Jun (1∶1000) for 1 h at room temperature. After 3 washes, the blots were subsequently incubated with a donkey anti-rabbit peroxidase-conjugated secondary antibody (1∶1000) for 1 h at room temperature. The blots were visualized with enhanced chemiluminescence and Kodak X-OMAT LS film (Eastman Kodak, Rochester, NY, USA).

### Immunofluorocytochemistry

SCC4 cells were cultured on 12-mm coverslips. After treatment with WISP-1, cells were fixed with 4% paraformaldehyde at room temperature. Thirty minutes later, 4% nonfat milk in PBS containing 0.5% Triton X-100 was added to the cells. The cells were then incubated with rabbit anti-c-Jun (1:100) and fluorescein isothiocyanate (FITC)-conjugated goat anti-rabbit secondary antibody (1:500; Leinco Technology Inc., St Louis, MO, USA) for 1 h, successively. FITC was detected using a Zeiss fluorescence microscope.

### Chromatin immunoprecipitation

Chromatin immunoprecipitation (ChIP) was performed as described [32]. DNA was immunoprecipitated using an anti-c-Jun antibody, extracted, purified, and resuspended in H_2_O. Immunoprecipitated DNA was amplified by PCR using the following primers: 5′-AGACCTTAGCGCGGTGTAGA-3′ and 5′-GCGACTCGAGGAGACGATGA-3′. PCR products were resolved by 1.5% agarose gel electrophoresis and visualized by ultraviolet light.

### Immunohistochemistry

A human OSCC tissue array was purchased from Biomax (Rockville, MD, USA; 26 cases for normal lingual tissue, 32 cases for grade 1 OSCC, 12 cases for grade 2 OSCC and 9 cases for grade 3 OSCC). The tissues were placed on glass slides, rehydrated, and incubated in 3% hydrogen peroxide to block the endogenous peroxidase activity. After trypsinization, sections were blocked by incubation in 3% BSA in PBS. The primary monoclonal mouse anti-human WISP-1 or ICAM-1 antibody was applied to the slides at a dilution of 1:50 and incubated at 4°C overnight. After being washed 3 times in PBS, the samples were treated with goat anti-mouse IgG biotin-labeled secondary antibody at a dilution of 1:50. Bound antibodies were detected with an ABC kit (Vector Laboratories). The slides were stained with chromogen diaminobenzidine, washed, counterstained with Delafield’s hematoxylin, dehydrated, treated with xylene, and mounted.

### Statistics

Data are presented as mean ± standard error of the mean (SEM). Statistical analysis between 2 samples was performed using the Student’s *t* test. Statistical comparisons of more than 2 groups were performed using one-way analysis of variance with Bonferroni’s post-hoc test. In all cases, p < 0.05 was considered significant.

## Results

### WISP-1 promotes migration in OSCC cells

**Figure 1:**
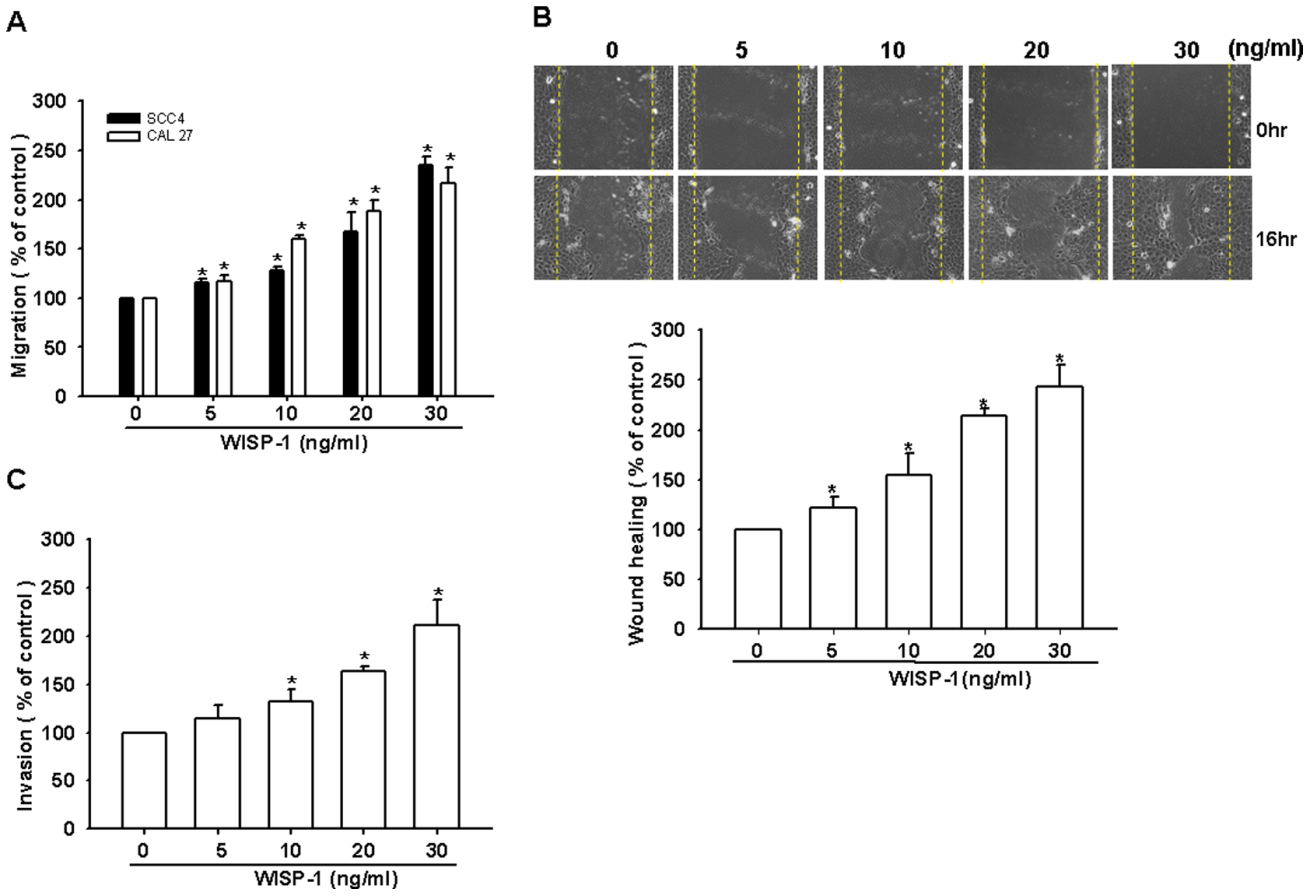
WNT1-inducible signaling pathway protein 1 (WISP-1) induces the migration activity of human oral squamous cell carcinoma (OSCC) cells. (A) Cells were incubated with various concentrations of WISP-1, and the *in vitro* migration activity was measured with the Transwell assay after 24 h (n = 5). (B) SCC4 cells were incubated with WISP-1 for 24 h, and the wound-scratching assay was performed (n = 4). (C) SCC4 cells were incubated with various concentrations of WISP-1, and the invasion activity was measured with the Transwell assay after 24 h (n = 5). Results are expressed as the mean ± standard error of the mean (SEM); *, p < 0.05 compared with the control; #, p < 0.05 compared with the WISP-1–treated group.

It has been reported that WISP-1 stimulates the directional migration and invasion of human cancer cells [10], [11], [33]. However, the effect of WISP-1 on the migration of OSCC cells is mostly unknown. To elucidate a link between WISP-1 expression and OSCC migration, we first examined the migratory activity of human OSCC cells using the Transwell assay. The stimulation of OSCC cells (SCC4 and CAL27 cells) with WISP-1 promoted cell migration (Fig. 1A). In addition, WISP-1 dose-dependently increased wound healing migration activity (Fig. 1B). Furthermore, WISP-1 enhanced the invasive activity of SCC4 cells through a Matrigel basement membrane matrix (Fig. 1F). Hence, WISP-1 promotes cell migration in human OSCC cells.

### Involvement of ICAM-1 in WISP-1–directed cell migration of OSCC cells

**Figure 2:**
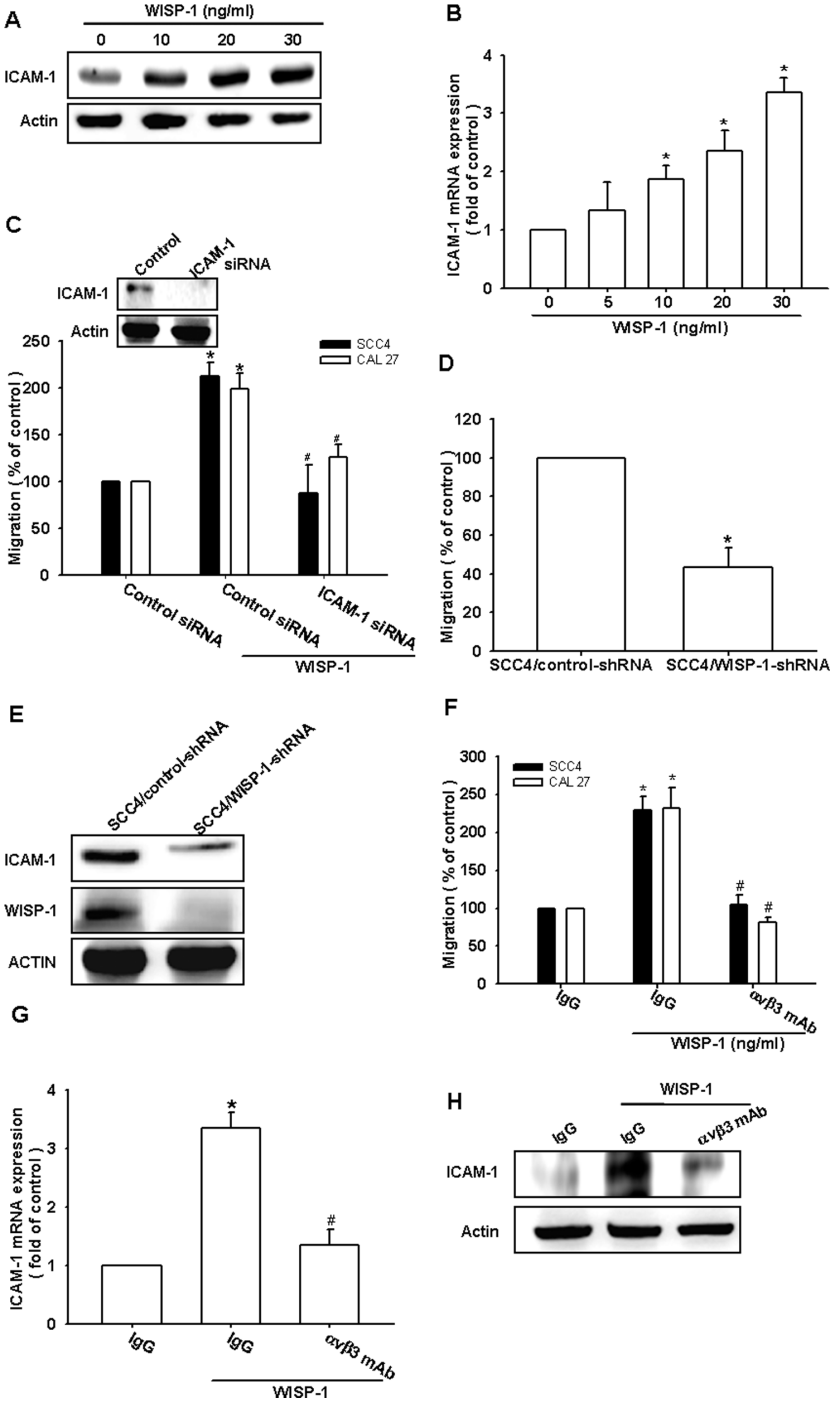
WISP-1 increases cell migration and intercellular adhesion molecule-1 (ICAM-1) expression through the αvβ3 integrin receptor. (A&B) SCC4 cells were incubated with WISP-1 for 24 h, and ICAM-1 expression was examined by western blotting and quantitative real-time polymerase chain reaction (qPCR) (n = 5). (C) Cells were transfected with ICAM-1 small interfering RNA (siRNA) for 24 h, followed by stimulation with WISP-1. The *in vitro* migration activity was measured with the Transwell assay (n = 4). (D&E) The protein levels and migratory activity of WISP-1 and ICAM-1 in SCC4/control short hairpin RNA (shRNA) and SCC4/WISP-1 shRNA cells were examined by western blotting and the Transwell assay (n = 4). (F–H) Cells were pretreated with αvβ3 monoclonal antibody (mAb) (10 µg/mL) for 30 min followed by stimulation with WISP-1. The *in vitro* migration activity and ICAM-1 expression were measured with the Transwell assay, qPCR, and western blotting (n = 5). Results are expressed as the mean ± SEM; *, p < 0.05 compared with the control; #, p < 0.05 compared with the WISP-1–treated group.

ICAM-1 upregulation has been reported to mediate the migration and metastasis of OSCC cells [34]. We therefore examined whether ICAM-1 was involved in WISP-1–induced migration of OSCC cells. The stimulation of cells with WISP-1 increased ICAM-1 protein and mRNA expression in a dose-dependent manner (Fig. 2A & B). Next, we used ICAM-1 siRNA to investigate whether ICAM-1 was involved in WISP-1–mediated cell migration. Transfecting cells with ICAM-1 siRNA markedly inhibited ICAM-1 expression and WISP-1–induced cell migration (Fig. 2C). To further confirm that WISP-1 mediates cell migration and ICAM-1 expression in human OSCC cells, SCC4 cells expressing WISP-1 shRNA were established. WISP-1 expression in stable transfectants was compared by western blotting. The expression of WISP-1 was dramatically inhibited in SCC4/WISP-1 shRNA cells (Fig. 2E). However, the knockdown of WISP-1 did not affect SCC4 cell growth (data not shown). The migratory ability of these transfectants was then analyzed using a Transwell migration assay. The knockdown of WISP-1 expression inhibited the migratory ability of SCC4 cells (Fig. 2D). In addition, WISP-1 knockdown also reduced ICAM-1 expression in SCC4 cells (Fig. 2E). These results indicated that WISP-1 increased cell migration through the upregulation of ICAM-1 in human OSCC cells.

WISP-1 is known to affect cell functions by binding to the cell-surface integrin αvβ3 receptor [35]. Pretreating the cells for 30 min with anti-αvβ3 monoclonal antibody (mAb) significantly reduced the WISP-1–increased cell migration and ICAM-1 expression (Fig. 2F–H). Thus, WISP-1 increased cell migration and ICAM-1 expression in human OSCC cells via the integrin αvβ3 receptor.

### Involvement of ASK1 in WISP-1–induced migration and ICAM-1 expression

**Figure 3:**
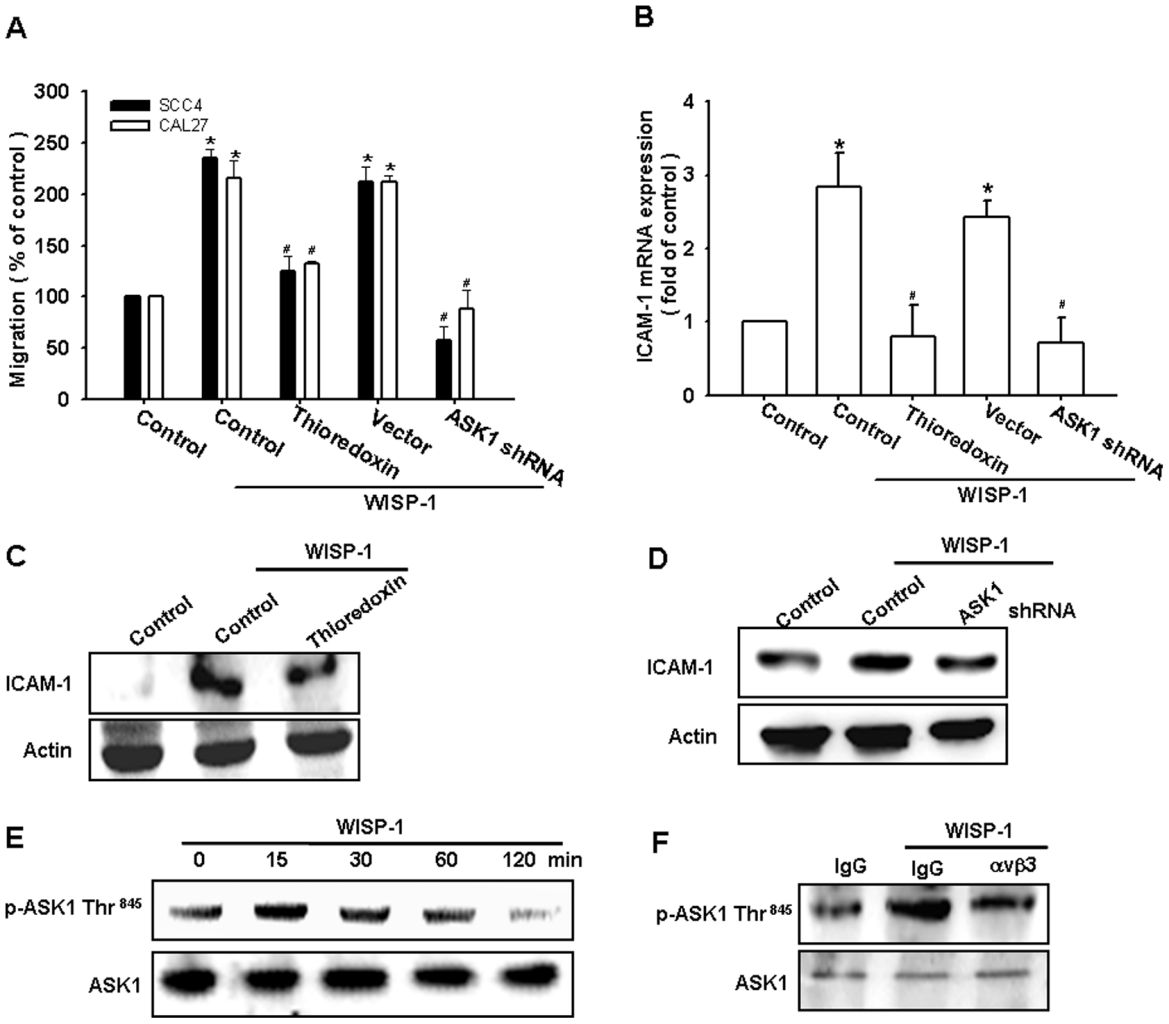
Apoptosis signal-regulating kinase 1 (ASK1) is involved in WISP-1–induced migration and ICAM-1 expression. (A–D) Cells were pretreated for 30 min with thioredoxin (200 ng/mL) or transfected with ASK1 shRNA for 24 h and stimulated with WISP-1. The *in vitro* migration and ICAM-1 expression were measured by the Transwell assay, qPCR, and western blotting (n = 5). (E) SCC4 cells were incubated with WISP-1 for the indicated time intervals, and ASK1 phosphorylation was examined by western blotting (n = 4). (F) SCC4 cells were pretreated for 30 min with αvβ3 mAb and stimulated with WISP-1 for 15 min; ASK1 phosphorylation was determined by western blotting (n = 4). Results are expressed as the mean ± SEM; *, p < 0.05 compared with the control; #, p < 0.05 compared with the WISP-1–treated group.

ASK1 is a member of the MKKK family, and is a part of the mitogen-activated protein kinase pathway [36]. ASK1 was found to be involved in cancer migration and metastasis [37]. We therefore hypothesized that ASK1 may be involved in the WISP-1–directed cell migration activity in OSCC cells. Our results showed that the WISP-1–induced migration ability and ICAM-1 upregulation of OSCC cells were greatly reduced by pretreating the cells with the ASK1 inhibitor thioredoxin (Fig. 3A–C). In addition, the transfection of cells with ASK1 shRNA also inhibited WISP-1–induced motility and ICAM-1 expression (Fig. 3A–D). We next directly measured the phosphorylation of ASK1 at Thr^845^ in response to WISP-1. Stimulating SCC4 cells with WISP-1 increased ASK1 phosphorylation at Thr^845^ (Fig. 3E). In addition, pretreating the cells with integrin αvβ3 mAb abolished the WISP-1–enhanced ASK1 phosphorylation (Fig. 3F). Thus, WISP-1 appears to act through integrin αvβ3 and the ASK1-dependent signaling pathway to enhance cell migration and ICAM-1 expression in human OSCC cells.

### The JNK and p38 signaling pathways are involved in the WISP-1–mediated ICAM-1 upregulation and cell motility of OSCC cells

**Figure 4:**
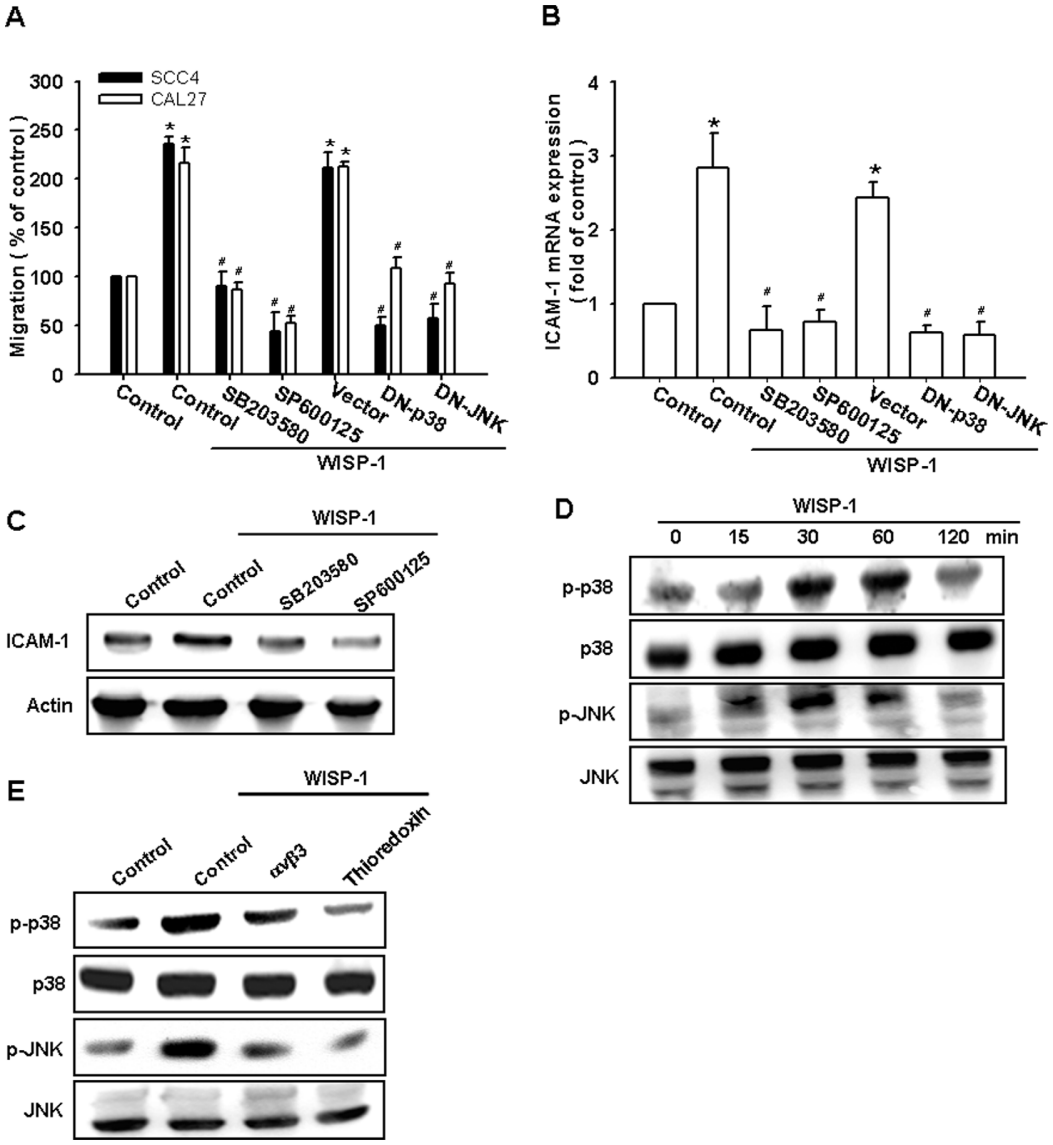
WISP-1 increases cell motility and ICAM-1 expression through the c-Jun N-terminal protein kinase (JNK) and p38 pathways. (A–C) Cells were pretreated for 30 min with SB203580 (10 µM) and SP600125 (10 µM) or transfected with dominant negative (DN) mutants of p38 and JNK for 24 h followed by stimulation with WISP-1. The *in vitro* migration and ICAM-1 expression were measured by the Transwell assay, qPCR, and western blotting (n = 5). (D) SCC4 cells were incubated with WISP-1 for the indicated time intervals, and p38 and JNK phosphorylation was examined by western blotting (n = 4). (E) SCC4 cells were pretreated for 30 min with αvβ3 mAb or thioredoxin for 30 min followed by stimulation with WISP-1 for 60 min, and JNK and p38 phosphorylation was determined by western blotting (n = 5). Results are expressed as the mean ± SEM; *, p < 0.05 compared with the control; #, p < 0.05 compared with the WISP-1–treated group.

ASK1 belongs to the MKKK family and activates the JNK and p38 pathways via MAPK kinase (MKK)4/7 and MKK3/6, respectively [23]. We next examined the role of JNK and p38 in mediating WISP-1–induced cell migration and ICAM-1 expression by using the specific JNK inhibitor SP600125 and p38 inhibitor SB203580. The pretreatment of cells for 30 min with SP600125 and SB203580 or transfection of cells for 24 h with mutant JNK and p38 reduced WISP-1–induced increases in cell motility and ICAM-1 expression (Fig. 4A–C). To directly confirm the crucial role of JNK and p38 in WISP-1–mediated cell migration, we measured the level of JNK and p38 phosphorylation in response to WISP-1. The stimulation of SCC4 cells with WISP-1 resulted in a time-dependent phosphorylation of JNK and p38 (Fig. 4D). We next evaluated the relationship between αvβ3 integrin, ASK1, and JNK/p38 in the WISP-1–mediated signaling pathway. Incubating the cells with integrin αvβ3 mAb or thioredoxin diminished the WISP-1–increased p38 and JNK phosphorylation (Fig. 4E). Based on these results, WISP-1 appears to act via the αvβ3 integrin receptor and the ASK1 and JNK/p38-dependent signaling pathways to enhance cell migration and ICAM-1 expression in human OSCC cells.

### Involvement of AP-1 in WISP-1–induced cell migration and ICAM-1 expression

**Figure 5:**
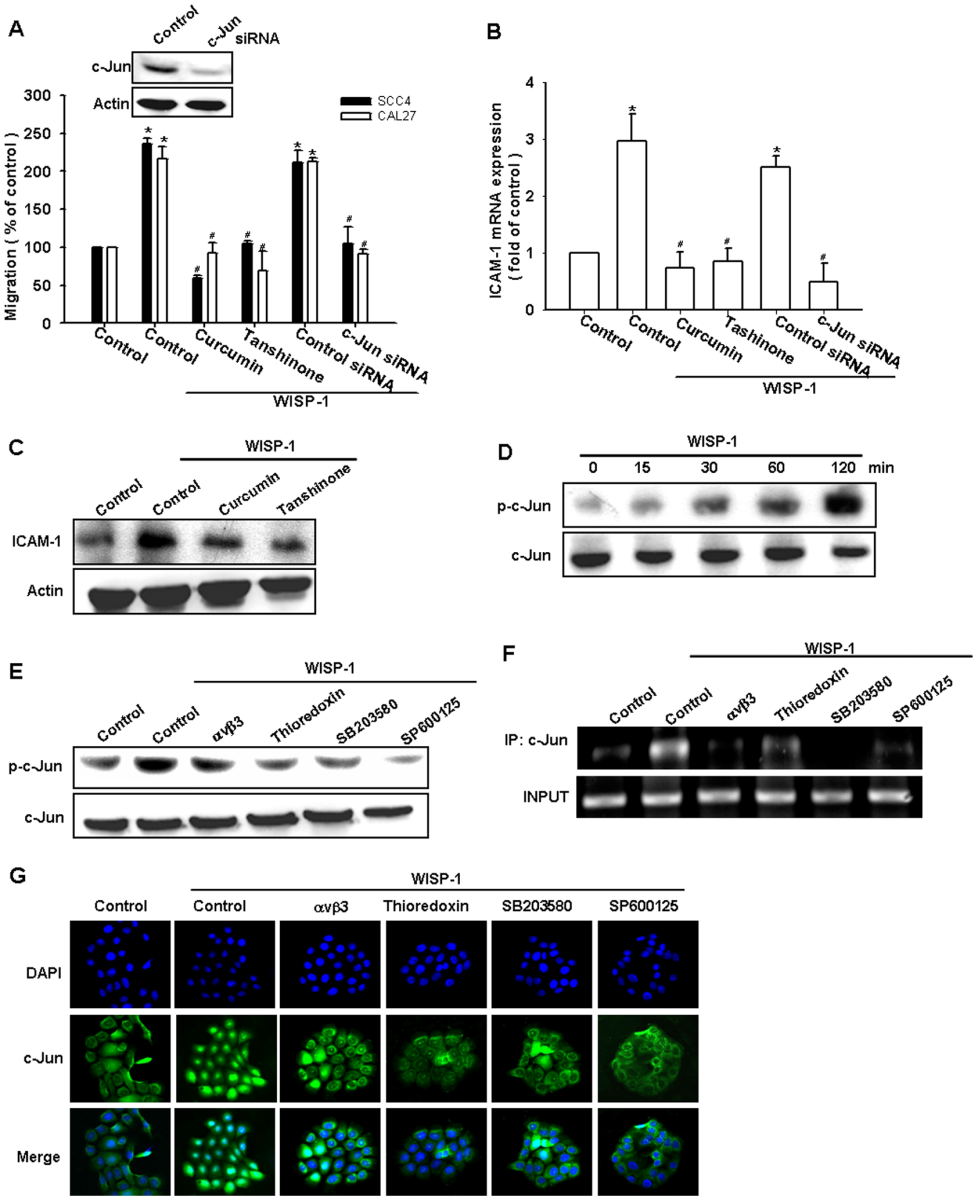
Activator protein 1 (AP-1) is involved in WISP-1–mediated migration in human OSCC cells. (A–C) Cells were pretreated for 30 min with curcumin (10 μM) and tanshinone (10 µM) or transfected for 24 h with c-Jun siRNA followed by stimulation with WISP-1 for 24 h. The *in vitro* migration and ICAM-1 expression were measured by the Transwell assay, qPCR, and western blotting (n = 5). (D) SCC4 cells were incubated with WISP-1 for the indicated time intervals, and c-Jun phosphorylation was examined by western blotting (n = 4). (E–G) SCC4 cells were pretreated for 30 min with αvβ3 mAb, thioredoxin, SB203580, or SP600125 for 30 min followed by stimulation with WISP-1 for 120 min. The c-Jun phosphorylation, c-Jun binding to the AP-1 element, and c-Jun translocation into the nucleus was determined by western blotting, chromatin immunoprecipitation, and immunofluorocytochemistry (n = 5). Results are expressed as the mean ± SEM; *, p < 0.05 compared with the control; #, p < 0.05 compared with the WISP-1–treated group.

As previously mentioned, AP-1 transactivation is involved in cell migration and ICAM-1 expression in human OSCC cells [38]. To examine the role of the AP-1-binding site in WISP-1–mediated cell motility and ICAM-1 expression, we used the AP-1 inhibitors (curcumin and tanshinone). Pretreating the cells with curcumin and tanshinone reduced WISP-1–induced cell migration and ICAM-1 expression (Fig. 5A–C). AP-1 activation was further evaluated by the analysis of c-Jun phosphorylation, ChIP, and c-Jun translocation into the nucleus. The transfection of cells with c-Jun siRNA suppressed WISP-1–induced cell migration and ICAM-1 expression (Fig. 5A & B). Incubating the cells with WISP-1 promoted the time-dependent phosphorylation of c-Jun (Fig. 5D). In contrast, pretreating the cells with integrin αvβ3 mAb, thioredoxin, SP600125, or SB203580 abolished WISP-1–mediated c-Jun phosphorylation (Fig. 5E).

The AP-1 binding site between -284 and -279 is important for the activation of the ICAM-1 gene [39]. We next investigated whether c-Jun binds to the AP-1 element in the ICAM-1 promoter after WISP-1 stimulation. The *in vivo* recruitment of c-Jun to the ICAM-1 promoter (–346 to –24) was assessed by ChIP. The *in vivo* binding of c-Jun to the AP-1 element of the ICAM-1 promoter occurred after WISP-1 stimulation (Fig. 5F). The binding of c-Jun to the AP-1 element by WISP-1 was attenuated by the integrin αvβ3 mAb, thioredoxin, SP600125, and SB203580 (Fig. 5F). In addition, integrin αvβ3 mAb, thioredoxin, SP600125, and SB203580 also reduced the WISP-1–increased c-Jun accumulation in the nucleus (Fig. 5G). Taken together, these data suggest that the activation of integrin αvβ3, ASK1, and JNK/p38 are required for WISP-1–induced AP-1 activation in human OSCC cells.

### WISP-1 and ICAM-1 expression correlates with the tumor stage of patients with OSCC

**Figure 6:**
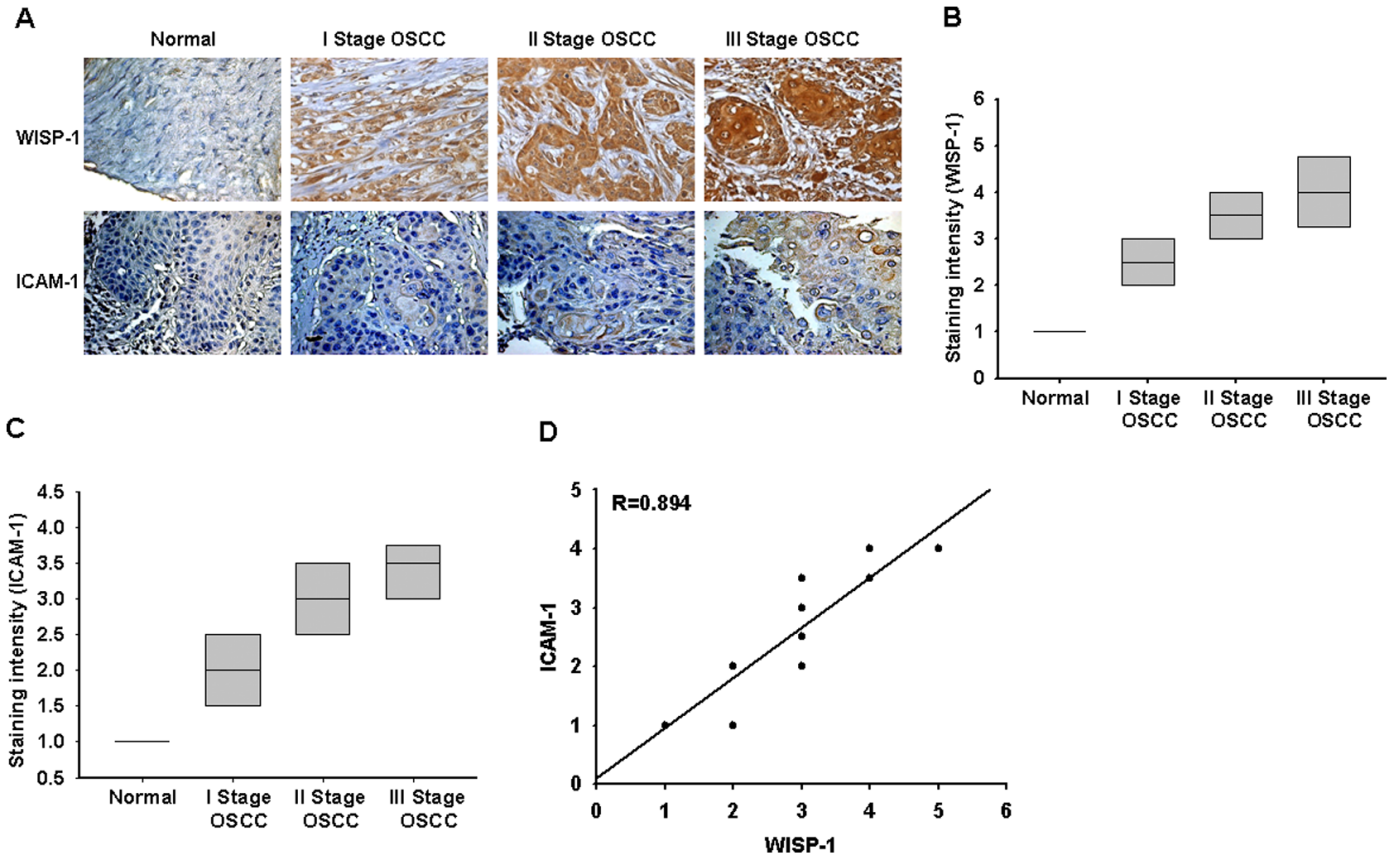
WISP-1 and ICAM-1 expression correlates with the tumor stage of patients with OSCC. Immunohistochemistry of WISP-1 (A&B) and ICAM-1 (A&C) expression in normal and OSCC tissue. The correlation and quantitative data are shown in (D).

To determine the clinical significance of WISP-1 and ICAM-1 expression in patients with cancer, we analyzed samples from OSCC patients by immunohistochemical staining. The expression of WISP-1 and ICAM-1 in OSCC patients was significantly higher than that in healthy individuals (Fig. 6A–C). In addition, the high level of WISP-1 expression correlated strongly with ICAM-1 expression and tumor stage (Fig. 6A–D). The quantitative data also show that the expression of WISP-1 is correlated with the expression of ICAM-1 in human OSCC patients (Fig. 6D). Taken together, these results indicate that WISP-1 and ICAM-1 expression correlates with tumor stage in patients with OSCC.

## Discussion

The elucidation of the molecular biology of cancer cells in recent years has identified various molecular pathways that are altered in different cancers. This information is currently being exploited to develop potential therapies that target molecules in these pathways. Although the mechanism of metastasis is a complicated and multistage process; however, our study showed that WISP-1 induces cell migration and the expression of ICAM-1 in human OSCC cells. In addition, we also provided the evidence that ICAM-1 acts as a crucial transducer of cell signaling, regulating cell migration, and WISP-1 acts as a critical mediator of the metastasis activity of OSCC cells in the tumor microenvironment. In the current study, we found that the high level of WISP-1 expression correlates strongly with ICAM-1 expression and tumor stage in OSCC patients. These data implicated that WISP-1 is mediated the carcinogenesis but not metastasis in OSCC. Because, these clinical samples were obtained from Biomax and they didn’t provided more detail information about tumors metastasize. Therefore, we can’t answer whether high level WISP-1 correlates with high metastasis *in vivo*. Nevertheless, our *in vitro* results are enough to provide the evidence that WISP-1 induced OSCC migration.

ICAM-1 is upregulated in response to a variety of cytokines and is associated with inflammatory and immune responses [17]. Several lines of evidence in this study show that, in addition to its role in leukocyte adhesion and cancer cell invasion [40], [41], ICAM-1 plays an important role in WISP-1–mediated cancer metastasis. First, WISP-1 promoted the mRNA and protein expression of ICAM-1. Second, ICAM-1 siRNA significantly reduced WISP-1–mediated cell motility. Third, SCC4/WISP-1 shRNA cells showed a greater reduction in migration and ICAM-1 expression than SCC4/control shRNA cells. Although ICAM-1 was reported to be associated with the cell motility of OSCC cells, we provide evidence that ICAM-1 is a downstream effector in WISP-1–increased metastasis of OSCC.

ASK1 is a member of the MKKK family and is part of mitogen-activated protein kinase pathway. ASK1 activity is regulated by multiple mechanisms including phosphorylation and interactions with various proteins, and it is also an upstream molecule of the JNK and p38 pathways, which are involved in the regulation of gene expression [42]. Phosphorylation at Ser^845^ is essential for ASK1 activation [43]. In this study, we found that WISP-1 enhanced ASK1 phosphorylation at Thr^845^. In addition, stimulating cells with WISP-1 also increased the JNK and p38 phosphorylation. Furthermore, the pretreatment of cells with either integrin αvβ3 mAb or ASK1 inhibitor reduced WISP-1–promoted JNK and p38 phosphorylation. We also found that integrin αvβ3 mAb, ASK1, JNK, and p38 inhibitor blocked WISP-1–increased cell motility and the expression of ICAM-1. This was further confirmed by the result that both ASK1 shRNA and mutant JNK and p38 inhibited the WISP-1–enhanced cell migration and ICAM-1 expression. Thus, our results provide evidence that WISP-1 upregulates ICAM-1 expression and cell migration in human OSCC cells via the integrin αvβ3, ASK1, and JNK/p38 signaling pathways. This study is not the first time to expose the ASK1 and JNK/p38 pathways in cancer metastasis. Lin et al., has been reported that ASK1-dependent JNK/p38 activation is involved in IL-6-induced angiogenesis and metastasis in osteosarcomas [25]. On the other hand, BDNF induced chondrosarcoma metastasis also through ASK1-dependent JNK/p38 pathway [32]. However, whether this pathway is common in cancer metastasis needs further investigation.

The Jun and Fos transcription factor families bind to the AP-1 sequence. These nuclear proteins interact with the AP-1 site as Jun homodimers or Jun-Fos heterodimers that are formed by protein dimerization through their leucine zipper motifs. Here, we found that WISP-1 promoted c-Jun phosphorylation and translocation into the nucleus. In addition, WISP-1–mediated cell migration and ICAM-1 expression were abolished by c-Jun siRNA in human OSCC cells. Therefore, c-Jun activation is mediated by WISP-1–increased cancer metastasis and ICAM-1 expression. Furthermore, WISP-1 increased the binding of c-Jun to the AP-1 element within the ICAM-1 promoter, as shown by ChIP. The pretreatment of cells with integrin αvβ3 mAb, thioredoxin, SP600125, and SB203580 abolished the binding of c-Jun to the AP-1 element. These results indicate that WISP-1 may act through the integrin αvβ3, ASK1, JNK/p38, c-Jun, and AP-1 pathways to induce cell migration and ICAM-1 expression in human OSCC cells.

In conclusion, we present a molecular mechanism of WISP-1–induced migration of human OSCC cells by the upregulation of ICAM-1. WISP-1 increases ICAM-1 expression through αvβ3 integrin, ASK1, JNK/p38, and AP-1 signaling pathways and induces tumor migration.
